# ARETA (Alpine caRbon cyclE daTAset): a dataset on physical, chemical and isotopic data of Alpine groundwaters

**DOI:** 10.1038/s41597-025-06541-0

**Published:** 2026-03-24

**Authors:** Marco Donnini, Laura Melelli, Marino Vetuschi Zuccolini, Federica Fiorucci, Carlo Cardellini, Francesco Frondini

**Affiliations:** 1 CNR-IRPI (Consiglio Nazionale delle Ricerche – Istituto di Ricerca per la Protezione Idrogeologica), Perugia, Italy; 2 Università degli Studi di Perugia – Dipartimento di Fisica e Geologia, Perugia, Italy; 3 Università di Genova – Dipartimento di Scienze della Terra, dell’Ambiente e della Vita, Genova, Italy

**Keywords:** Carbon cycle, Water resources

## Abstract

In Europe 65% of drinking water and 25% of water for agricultural irrigation come from groundwaters. Thermal and mineral groundwaters have an important role in society, for well-being and for economic purposes. Although widespread Alpine aquifers are critically important and highly vulnerable, regional-scale quantitative and qualitative studies on these groundwater resources remain remarkably limited. In this work we compiled a geo-dataset named ARETA (Alpine caRbon cyclE daTAset), containing more than 3,000 chemical analyses of georeferenced spring waters obtained both from the literature (technical reports, scientific publications, books, and other bibliographic sources) and from unpublished data collected during 2011–2022 fieldworks. For fewer than 20% of spring waters, analysis of the isotopic composition of water and carbon were also included, as well as flow rate values. The ARETA dataset significantly advances knowledge by addressing key geographic and hydrogeochemical gaps within the Alpine chain. Its broad coverage makes it an invaluable resource, especially when integrated with other established databases for large-scale studies. The dataset is publicly available at Figshare^1^.

## Background & Summary

According to the European Environment Agency, in Europe 65% of drinking water and 25% of water for agricultural irrigation come from underground aquifers^2^. Thermal (high temperature) and mineral waters (high salinity content) have played an important role in society, both for well-being and for economic purposes, since Roman times. Within mineral waters, the brines (waters with high temperature and high saline concentration), have gained importance in recent years due to their high lithium content, since lithium is a strategic metal — especially for batteries in electric vehicles — for which worldwide demand is constantly increasing^3^.

The prevailing literature emphasizes that precipitation in the Alps preferentially generates surface runoff rather than deep infiltration^4^. Nevertheless, recent studies have refuted the long-held notion of negligible groundwater storage, indicating that aquifers are, in fact, widespread in the Alps^5^. The water storage capacity — found in glaciers, lakes, and aquifers — allows the Alps to function as a critical water tower, feeding major European river basins, including the Rhine, Danube, Po, and Rhone^5^, which are notable for their high discharge rates and extensive drainage areas. Consequently, the Alpine region is deemed highly sensitive to climate change regarding the stability and accumulation of deep-water resources^5^.

From a geomorphologic perspective, the Alps exhibit a strong topographic variability with extensive lowlands, deeply incised valleys, and mountain peaks higher than 4000 m. a.s.l.^6^. The Alps exhibit a strong relationship between physiography and climate^7,8^. Valley bottoms experience warmer temperatures and lower precipitation compared to surrounding higher elevations^9^, snowfall accumulates during the winter season and melts in the following season^10^, and glaciers currently cover an area of approximately 2050 km²^11^ representing roughly 1% of the total Alpine region^12^.

**Fig. 1:**
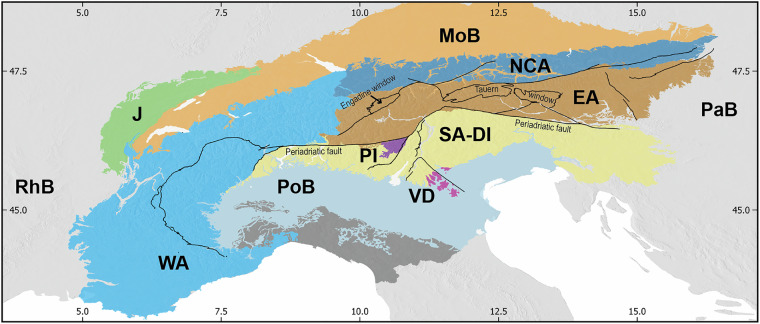
Schematic geological map of Alps derived from different studies^12–14,16–18^. WA = Western Alps, EA = Eastern Alps, NCA = Northern Calcareous Alps, SA-DI = Southern Alps-Dinarides, PI = Periadriatic Inclusions, VD = Volcanic Districts, J = Jura mountains, PoB = Po Basin, RhB = Rhone Basin, MoB = Molasse Basin PaB = Pannonian Basin.

The hydrogeological characteristics of the Alps are remarkably heterogeneous, owing to their exceptionally complex geological configuration^12–15^. Figure 1 shows a schematic geological map of Alps derived from different studies^12–14,16–18^ highlighting the presence of the following geological domains: the Western Alps (WA), the Northern Calcareous Alps (NCA), the Eastern Alps (EA), the Southern Alps that are partially continuous to the Dinarides (SA-DI), the Periadriatic Inclusions (PI), and the Volcanic Districts (VD). The Alps are circumscribed by the Rhone Basin (RhB) to the west, the Jura Mountains (J) define the north-western boundary, the Molasse Basin (MoB) to the north, the Pannonian Basin (PaB) to the east, and the Po Basin (PoB) to the south. The black lines on the map represent major tectonic lineaments, such as the Periadriatic fault and those defining the Engadine and Tauern tectonic windows.

Despite the hydrological significance and climate vulnerability of the region, detailed hydrogeological studies — both qualitative and quantitative — remain rare across the vast Alpine area (over 200,000 km^2^), which encompasses six different countries (France, Italy, Switzerland, Germany, Austria, and Slovenia).

While existing literature provides valuable specialized datasets, focusing on thermal spring characteristics (estimating deep groundwater flow from 311 Alpine thermal springs)^19^ and on the karst springs discharge (the World Karst Spring hydrograph WoKaS^20^ which contains information regarding ∼400 karst springs across the world) comprehensive physical, chemical, and isotopic datasets for both freshwater and mineral waters in the region remain limited.

To address this gap we developed and compiled a geo-dataset named ARETA (Alpine caRbon cyclE daTAset), which includes physical, chemical, and isotopic data for more than 3,000 Alpine spring waters, including mineral, thermal and freshwater springs. The data were sourced from both existing literature and sampling campaigns conducted by the authors between 2011 and 2022. Particular attention was given to including data from high-discharge springs, as well as mineral and thermal waters. A significant portion of the 2011–2022 sampling efforts specifically targeted waters with these characteristics (high discharge, high temperatures, and high salinity) for which literature data were scarce.

## Methods

ARETA dataset provides detailed physical, chemical and isotopic information for Alpine spring waters. Physical data include discharge (*Q*), temperature (*T*), *pH*, redox potential (*Eh*), and electrical conductivity (*EC*). Chemical data comprise the composition of the major soluble ions HCO_3_^-^, CO_3_^2-^, Ca^2+^, Mg^2+^, Na^+^, K^+^, Cl^-^, NO_3_^-^, SO_4_^2-^. Isotopic data include the water stable isotopes δ^2^H (Deuterium, or δD) and δ^18^O; the carbon stable isotopes δ^13^C and the unstable carbon isotope ^14^C (radiocarbon) of the Total Dissolved Inorganic Carbon (*TDIC*); and the composition of Tritium (^3^H).

*Q*, *T*, *Eh*, and *EC* are expressed, respectively, in L/s, °C, mv, μS/cm, while *pH* is dimensionless. The major soluble ions are expressed in mg/L and in meq/L. δD and δ^18^O are expressed in delta notation (δ‰) relative, respectively, to Vienna Standard Mean Ocean Water (V-SMOW), and to Vienna Pee Dee Belemnite (V-PDB). ^14^C is expressed in units of percent modern carbon (pmC). ^3^H is expressed in Tritium Units (T.U.), where 1 T.U. corresponds to 0.118 Bequerel per liter^21,22^.

Physical, chemical and isotopic data of Alpine spring waters were collected from 2009 as part of a PhD thesis at the University of Perugia (Italy) funded by CNR-IRPI (*Consiglio Nazionale delle Ricerche – Istituto di Ricerca per la Protezione Idrogeologica*), and collection concluded in 2023 during post-doctoral activities. These data were subsequently incorporated into a relational geo-dataset, which was developed to hold global Earth’s carbon cycle information and served as a supporting framework for the Connect4Carbon project (PRIN2017 Project – n. 2017LMNLAW, https://prin2017.wixsite.com/connectforcarbon). The integration of the data effectively served as the initial validation, a pilot phase, for the newly established geo-dataset structure.

The dataset structure was developed to store physical, chemical, and isotopic data of spring waters as a supporting tool for carbon balance estimates from regional aquifers; consequently, the current study does not account for the hydro-geochemical temporal variability observed at the individual sampling points. This methodology is appropriate given the scope of the investigation, which focuses on long-term geodynamic processes across vast areas, where temporal variations are less relevant than for short-scale, transient phenomena (e.g., volcanic eruptions or rapid fumarolic changes). For this reason, the dataset is structured with one-to-one relationships to ensure that each sampling point corresponds to a univocal hydro-geochemical measurement, since we are not interested in investigating the hydro-geochemical variation in time of the single sampling points.

When multiple information regarding physical, chemical, and isotopic data on a specific spring were available, we selected the most recent information available in the literature. This choice is justified by the primary focus on long-term geodynamic processes at a regional scale, where short-term (e.g., seasonal) hydro-geochemical variability is considered secondary. Consequently, retaining only the more recent measurements — which generally benefits from improved analytical techniques and represent a small minority of the dataset — was deemed the most pragmatic approach, as the statistical effort of averaging or weighted estimation would not significantly alter the final modelling results.

For newly collected samples, we applied a sampling procedure widely documented and accepted in the scientific literature^12,23–25^. For each sample, *T*, *EC*, *pH*, *Eh*, and alkalinity (HCO_3_^-^ concentration) were measured in the field. Specifically, *T*, *EC*, *pH*, and *Eh* were determined using a multi-parameter portable meter, while alkalinity was determined by acid titration with HCl 0.1 N using methyl orange as indicator. Water samples for chemical analyses for the concentration of major soluble ions (Ca^2+^, Mg^2+^, Na^+^, K^+^, Cl^-^, NO_3_^-^, SO_4_^2-^) were filtered through 0.45 μm filters and collected in two polyethylene bottles with a volume of 100 and 50 mL. The 50 mL aliquot was acidified with 0.5 mL of concentrated suprapure HCl to stabilize the sample and prevent algal growth and carbonate precipitation. For the determination of the stable water isotopes (δD, δ^18^O) a raw sample was collected in a 100 mL polyethylene bottle. Samples for the analysis of δ^13^C in the *TDIC* were collected in 1000 mL polyethylene bottles where solid SrCl_2_ and NaOH were added to precipitate the dissolved inorganic carbon as strontium carbonate (SrCO_3_). The carbonate precipitate was recovered in the laboratory by filtration and rinsed with CO_2_-free distilled water.

Major ion concentrations were determined at the University of Perugia laboratory. Ca^2+^ and Mg^2+^ concentrations were analysed in the acidified samples by atomic absorption (AA) flame spectrometry. Na^+^ and K^+^ concentrations were determined by atomic emission (AE) flame spectrometry using an Instrumentation Laboratory model AA/EE spectrophotometer 951. Cl^-^ and SO_4_²^−^ concentrations were analysed by ion chromatography using a Dionex DX-120 system. The detection limits are: 0.01 mg/L for Ca^2+^, Mg^2+^, Na^+^, K^+^, NO_3_^-^; and 0.03 mg/L for Cl_-_ and SO_4_^2-^.

The isotope analyses of δ^13^C, δD, and δ^18^O were performed at the Geochemistry Laboratory of INGV-OV (*Istituto Nazionale di Geofisica e Vulcanologia – Osservatorio Vesuviano*) in Napoli using a Finnigan Delta Plus XP continuous flow isotope ratio mass spectrometer coupled to a Gasbench II automated sample preparation device. Analytical uncertainties are ± 1‰ for δD, ± 0.08‰ for δ^18^O, and ± 0.06‰ for δ^13^C.

The information for populating the dataset derived from different sources is shown in Table 1.

**Table 1:** Data composing ARETA.

Dataset type	Dataset source	Type	Reference
Elevation data	GTOPO30DEM	Raster	https://earthexplorer.usgs.gov; 10.5066/F7DF6PQS
Administrative data	GISCO dataset	Vector	https://www.eea.europa.eu
Geo-lithological data	GLiM	Vector	^26^
	Geo-Lim	Vector	^27^
Physical, chemical, and isotopic spring data	Literature data	Different type	See Table 2
	New sampling campaigns	Vector and spreadsheet	

The topographic parameters for each sampling point were derived from the Digital Elevation Model (DEM) GTOPO30 released worldwide in raster format with horizontal grid spacing of 30 arc seconds (~1 km) (10.5066/F7DF6PQS; https://earthexplorer.usgs.gov).

The administrative boundaries are useful data for the localization of sampling points. To have a common dataset for the European area, we used the GISCO dataset (https://www.eea.europa.eu) that provides a medium-scale layer for regional administrative boundaries (NUTS) for the entire EU territory. The hierarchy of administrative land accounting units allows the analysis of the data at various scales, from NUTS 3 (province level) to NUTS 0 (country level).

The Global Lithological Map database v1.1 (GLiM^26^) has been used as a common dataset for deriving the geological information around the sampling points. We also considered the Geo-Lithological Map for Central Europe Geo-LiM^27^, elaborated at 1:1,000,000 scale, that better discriminates carbonate rocks.

The physical, chemical, and isotopic spring data refer to 3,381 sampling points sourced from various types of documents and activities (see Table 2). Overall, the data were compiled from 39 technical reports (33%), 24 scientific papers (27%), 5 books (13%), and 2 conference papers (5%). The remaining sources include national authorities (13%), websites (4%), new data published in this work (3%), and local authorities and personal communications (2%).

**Table 2:** Summary of data sources for the spring water analysis, indicating the number of sampling points and corresponding references.

Source	Number of data records	References
Technical reports	1,121	^31–67^
Scientific papers	904	^68–91^
Books	449	^92–96^
National authorities	442	https://www.bafu.admin.ch/bafu/en/home.html^97^; https://wasser.umweltbundesamt.at/h2odb/^98^
Conference papers	169	^99,100^
Web sites	140	http://www.acqua2o.it (last access: November 2025); http://www.mineralwaters.org (last access: August 2012); http://mineralwaters.geo.uu.nl/world.php (last access: November 2025); http://www.acqueitaliane.fondazioneamga.org (last access: August 2012)
New data published in this work	109	Not applicable
Local authorities	40	ARPA Lombardia (https://www.arpalombardia.it/); ARPA Piemonte (https://www.arpa.piemonte.it/)
Personal communications	7	Not applicable

## Data Records

The relational geo-dataset ARETA, which contains the Alpine spring data, is compliant with the use of Geographic Information Systems (GIS) software, it is reproducible worldwide, and it is available for download at 10.6084/m9.figshare.29850941^1^.

**Fig. 2:**
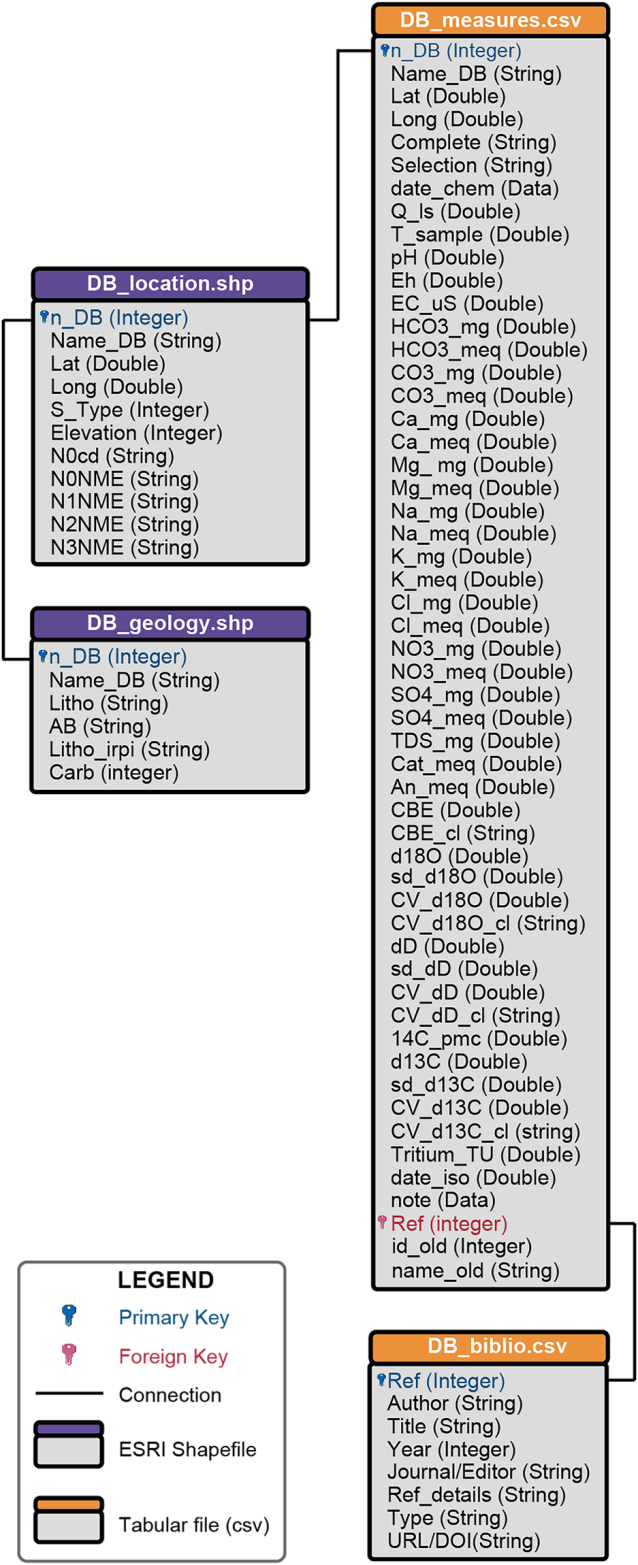
Physical structure of ARETA. See text for explanation.

Dataset ARETA, is composed of two shapefiles, *DB_location.shp* and *DB_geology.shp*, and two comma-separated values (CSV) files: *DB_measures.csv*, and *DB_biblio.csv* (Fig. 2). The attribute tables of the shapefiles and the data in the CSV files are linked through a one-to-one relationship using a key field, ensuring that each sampling point is uniquely matched to its corresponding hydrogeochemical measurement.

In the data model, *DB_location.shp* serves as the primary entity, storing core attributes (coordinates, elevation, and administrative boundaries) for each sampling point (see Table 3). Each sampling point is associated a univocal number (*n_DB*) that represents the primary key functional to join each tuple (i.e. record) of the files (vector and tabular) of the dataset. The latitude and longitude values are expressed in WGS84, decimal degrees. The elevation value was assigned from the corresponding cell in GTOPO30 DEM (10.5066/F7DF6PQS; https://earthexplorer.usgs.gov). The original values in DEM with decimal numbers, were rounded since integer numbers have been preferred. The administrative boundaries came from the nomenclature of territorial units for statistics (NUTS) released by Eurostat (www.eea.europa.eu).

**Table 3:** Fields of the DB_location.shp table.

Field	Type	Description
n_DB	Integer	Primary key field. The value represents the univocal number associated to the sampling point
Name_DB	String	Name of the sampling point assigned manually
Lat	Double	Latitude coordinate (WGS84 decimal degrees) assigned from GTOPO30DEM
Long	Double	Longitude coordinate (WGS84 decimal degrees) assigned from GTOPO30DEM
S_Type	Integer	Sample type (0 = unknown; 1 = natural spring; 2 = captured spring; 3 = thermal spring; 4 = resurgence spring; 5 = lake river waterfall; 6 = bottled mineral water; 7 = well; 8 = cave; 9 = tunnel gallery; 10 = other
Elevation	Integer	Elevation (m a.s.l.) rounded values from the global digital elevation model GTOPO30 (10.5066/F7DF6PQS; https://earthexplorer.usgs.gov)
N0CD	String	Acronym of the zero order of administrative boundary from the territorial units for statistics (NUTS) released by Eurostat (e.g. IT) (www.eea.europa.eu)
N0NME	String	Extended name of the zero order of administrative boundary from the territorial units for statistics (NUTS) released by Eurostat (e.g. IT ITALY) (www.eea.europa.eu)
N1NME	String	First order of administrative boundary from the territorial units for statistics (NUTS) released by Eurostat (e.g. ITD NORD-EST) (www.eea.europa.eu)
N2NME	String	Second order of administrative boundary from the territorial units for statistics (NUTS) released by Eurostat (e.g. ITD3 Veneto) (www.eea.europa.eu)
N3NME	String	Third order of administrative boundary from the territorial units for statistics (NUTS) released by Eurostat (e.g. ITD33 Belluno) (www.eea.europa.eu)

The *DB_geology.shp* file stores information of the lithology in correspondence of the sampling point considering both the most recent Global lithological map GLiM^26^ and the geo-lithological map for Central Europe Geo-LiM^27^ (see Table 4) that, differently from GLIM, takes into account the chemical and mineralogical composition of the outcropping rocks, fundamental to investigate the interactions between rocks and groundwaters. As shown in Fig. 2, *DB_geology.shp* is joined to *DB_location.shp* by the primary key *n_DB*.

**Table 4:** Fields of the DB_geology.shp table.

Field	Type	Description
n_DB	Integer	Primary key field. The value represents the univocal number associated with the sampling point.
Name_DB	String	Name of the sampling point
Litho	String	Lithological class from GLIM^26^. Evaporites; Ice and Glaciers; Metamorphics; No Data; Acid plutonic rocks; Basic plutonic rocks; Intermediate plutonic rocks; Pyroclastics; Carbonate sedimentary rocks; Mixed sedimentary rocks; Siliciclastic sedimentary rocks; Unconsolidate sediments; Acid volcanic rocks; Basic volcanic rocks; Intermediate volcanic rocks; Water bodies; Precrambrian rocks; Complex lithology; Alluvial deposits; Mafic metamorphics mentioned; Dune sands; Greenstone mentioned; Laterites; Loess; Mixed grain size; Organic sediment; (Pure) carbonate; Pyroclastics mentioned; Fine grained; Coarse grained; Black shale mentioned; Chert mentioned; Fossil plant organic material mentioned; Subordinate evaporites mentioned; Reduced – Iron minerals mentioned; Glacial influence mentioned; Metamorphic influence mentioned; Phosphorus – rich minerals mentioned; Subordinate plutonics mentioned; Pyrite mentioned; Subordinate sedimentary rocks mentioned; Subordinate unconsolidated sediments mentioned; Subordinate volcanic mentioned; Intensive weathering
AB	String	Lithological class code from GLIM^26^. ev = Evaporites; ig = Ice and Glaciers; mt = Metamorphics; nd = No Data; pa = Acid plutonic rocks; pb = Basic plutonic rocks; pi = Intermediate plutonic rocks; py = Pyroclastics; sc = Carbonate sedimentary rocks; sm = Mixed sedimentary rocks; ss = Siliciclastic sedimentary rocks; su = Unconsolidate sediments; va = Acid volcanic rocks; vb = Basic volcanic rocks; vi = Intermediate volcanic rocks; wb = Water bodies; pr = Precrambrian rocks; Cl = Complex lithology; ad = Alluvial deposits; am = Mafic metamorphics mentioned; ds = Dune sands; gr = Greenstone mentioned; la = Laterites; lo = Loess; mx = Mixed grain size; or = Organic sediment; pu = (Pure) carbonate; py = Pyroclastics mentioned; sh = Fine grained; ss = Coarse grained; bs = Black shale mentioned; ch = Chert mentioned; cl = Fossil plant organic material mentioned; ev = Subordinate evaporites mentioned; fe = Reduced – Iron minerals mentioned; gl = Glacial influence mentioned; mt = Metamorphic influence mentioned; ph = Phosphorus – rich minerals mentioned; pr = Subordinate plutonics mentioned; pt = Pyrite mentioned; sr = Subordinate sedimentary rocks mentioned; su = Subordinate unconsolidated sediments mentioned; vr = Subordinate volcanic mentioned; we = Intensive weathering
litho_irpi	String	Lithological class of Geo-LiM^27^. Pure carbonate; Mixed carbonate; Gypsum evaporite; Acid rocks; Mafic rocks; Intermediate rocks; Sandstones; Claystones; Peat; Metamorphic rocks
carb	Integer	0 = non-carbonate lithologies; 1 = carbonate lithologies according to the lithological classes of Geo-LiM^27^. Carbonate lithologies = Pure carbonate; Mixed carbonate; Gypsum evaporite. Non-carbonate lithologies = Acid rocks; Mafic rocks; Intermediate rocks; Sandstones; Claystones; Peat; Metamorphic rocks

The *DB_measures.csv* file stores the hydro-geochemical information that encompass physical, chemical, and isotopic data (see Table 5). In more detail, the physical data comprise *Q*, *T*, *pH*, *Eh*, and *EC*; the chemical data are the concentration of major soluble ions HCO_3_^-^, CO_3_^2-^, Ca^2+^, Mg^2+^, Na^+^, K^+^, Cl^-^, NO_3_^-^, SO_4_^2-^; and finally the isotopic data are the isotopic composition of water δ^18^O, δD and ^3^H, as well as the isotopic composition of *TDIC* (^14^C and δ^13^C). Moreover, the *DB_measures.csv* file also contains information regarding quality of chemical and isotopic analyses. Similarly to *DB_geology.shp*, also *DB_measures.csv* is linked to *DB_location.shp* through the primary key *n_DB* (see Fig. 2). Finally, *DB_measures.csv* contains the original identification name and number of the sampling point as mentioned by the authors of the bibliographic source.

**Table 5:** Fields of the DB_measure.csv table.

Field	Type	Description
n_DB	Integer	Primary key field. The value represents the univocal number associated to the sampling point
Name_DB	String	Name of the sampling point
Lat	Double	Latitude coordinate (WGS84 decimal degrees) assigned from GTOPO30DEM
Long	Double	Longitude coordinate (WGS84 decimal degrees) assigned from GTOPO30DEM
Complete	String	The field informs if the analyses are complete (value = YES) or are not complete (value = NO)
Selection	String	The field informs if the analyses are selected (value = YES) or not (value = NO)
date_chem	Data	The day/month/year when the sample has been collected for the chemical analyses
Q_ls	Double	Water discharge of the sampling point expressed in l/s
T_sample	Double	Temperature of the sampled point expresses in °C
pH	Double	pH of the sampled point
Eh	Double	Oxidation-reduction potential of the sampled point expressed in milliVolt (mV)
EC_uS	Double	Electrical conductivity of the sampled point expressed in μS/cm
HCO3_mg	Double	Bicarbonate concentration of the sampled point expressed in mg/l
HCO3_meq	Double	Bicarbonate concentration of the sampled point expressed in meq/l
CO3_mg	Double	Carbonate concentration of the sampled point expressed in mg/l
CO3_meq	Double	Carbonate concentration of the sampled point expressed in meq/l
Ca_mg	Double	Calcium concentration of the sampled point expressed in mg/l
Ca_meq	Double	Calcium concentration of the sampled point expressed in meq/l
Mg_mg	Double	Magnesium concentration of the sampled point expressed in mg/l
Mg_meq	Double	Magnesium concentration of the sampled point expressed in meq/l
Na_mg	Double	Sodium concentration of the sampled point expressed in mg/l
Na_meq	Double	Sodium concentration of the sampled point expressed in meq/l
K_mg	Double	Potassium concentration of the sampled point expressed in mg/l
K_meq	Double	Potassium concentration of the sampled point expressed in meq/l
Cl_mg	Double	Chloride concentration of the sampled point expressed in mg/l
Cl_meq	Double	Chloride concentration of the sampled point expressed in meq/l
NO3_mg	Double	Nitrate concentration of the sampled point expressed in mg/l
NO3_meq	Double	Nitrate concentration of the sampled point expressed in meq/l
SO4_mg	Double	Sulphate concentration of the sampled point expressed in mg/l
SO4_meq	Double	Sulphate concentration of the sampled point expressed in meq/l
Cat_meq	Double	Sum of the cation concentrations expressed in meq/l.
An_meq	Double	Sum of the anion concentrations expressed in meq/l
CBE	Double	Charge Balance Error
CBE_cl	String	Charge Balance Error quality class (low, medium, high)
TDS_mg	Double	Total Dissolved Solids expressed in mg/l
d18O	Double	δ^18^O composition of the sampled point
sd_d18O	Double	Standard deviation of the δ^18^O analysis of the sampled point
CV_d18O	Double	Coefficient of variation of the δ^18^O analysis of the sampled point
CV_d18O_cl	String	Coefficient of variation quality class of the δ^18^O analysis of the sampled point (low, medium, high)
dD	Double	δD (δ^2^H, Deuterium) composition of the sampled point
sd_dD	Double	Standard deviation of the δD (Deuterium) composition of the sampled point
CV_dD	Double	Coefficient of variation of the δD analysis of the sampled point
CV_dD_cl	String	Coefficient of variation quality class of the δD analysis of the sampled point (low, medium, high)
14 C_pmc	Double	^14^C (pMC percent Modern Carbon) composition of the sampled point
d13C	Double	δ^13^C composition of the sampled point
sd_d13C	Double	Standard deviation of the δ^13^C composition of the sampled point
CV_d13C	Double	Coefficient of variation of the δ^13^C analysis of the sampled point
CV_d13C_cl	String	Coefficient of variation quality class of the δ^13^C analysis of the sampled point (low, medium, high)
Tritium_TU	Double	^3^H composition expressed in Tritium Units (T.U.)
date_iso	Double	The day/month/year when the sample has been collected for the isotope analysis
note	Data	Annotations that cannot be included in the previous fields
Ref	Integer	Number code associated univocally to the literature source (or the other source)
id_old	Integer	The identification number of the sampling point as mentioned in the bibliographic source
name_old	String	The name of the sampling point as mentioned in the bibliographic source

The *DB_biblio.csv* files contain the bibliographic information on the data sources (see Table 6) and is linked to *DB_measures.csv* by the key *Ref* (foreign in *DB_measures.csv* and primary in *DB_biblio.csv*). In particular, *DB_biblio.csv* contains the complete information regarding the bibliographic source (author, year, journal/editor, reference details, typology, and URL/DOI) are stored in *DB_biblio.csv*.

**Table 6:** Fields of the DB_biblio.csv table.

Field	Type	Description
Ref	Integer	Primary key field to link the ref.csv table to the biblio.csv table
Authors	String	Authors of the document
Title	String	Title of the document
Year	Integer	Year of the document
Journal/Editor	String	Name of the journal or editor of the document
Ref_detail	String	Volume and pages
Type	String	Type of document
URL/DOI	String	Website or DOI or ISBN or ISSN

### Data Overview

A comprehensive summary of the ARETA dataset is provided in Table 7. Overall, the data demonstrates remarkable completeness, though the availability of individual parameters varies. Physical parameters, in particular, are well-represented: *T* and *pH* are available for the vast majority of samples, accounting for approximately 93% (3,138 points) and 92% (3,098 points), respectively. *EC* data are also common, available for 2,264 points (∼67%), while *Q* and *Eh* are less frequent, covering 1,481 (∼44%) and 719 (∼21%) points, respectively. Regarding chemical composition, completeness is exceptionally high: data on major soluble ions are included for 3,374 out of 3,381 total sampling points. The remaining seven points exclusively contain isotopic data (δD and δ^18^O), with one of these seven points also including δ^13^C and ^3^H values. Finally, the section concerning isotopic data is more heterogeneous: the dataset includes 601 analyses for stable water isotopes (δD and δ^18^O) and 114 sampling points for ^3^H. Pertaining to *TDIC*, δ^13^C data are available for 139 samples, while ^14^C is present in 56 points only.

**Table 7:** Number of sampling points containing physical, chemical, and isotopic analyses, grouped by general parameter and analysed sample matrix.

Sample matrix	General parameters	Specific parameter	Unit of measure	Number of sampling points
Water	Physical parameters	*Q*	L/s	1,481
		*T*	°C	3,138
		*pH*	dimensionless	3,098
		*Eh*	mv	719
		*EC*	μS/cm	2,264
	Chemical analyses	Major ions	mg/L and meq/L	3,374
	Isotopic analyses	δD	‰	601
		δ^18^O	‰	601
		^3^H	T.U.	114
*TDIC*		δ^13^C	‰	139
		^14^C	pmC	56

**Fig. 3:**
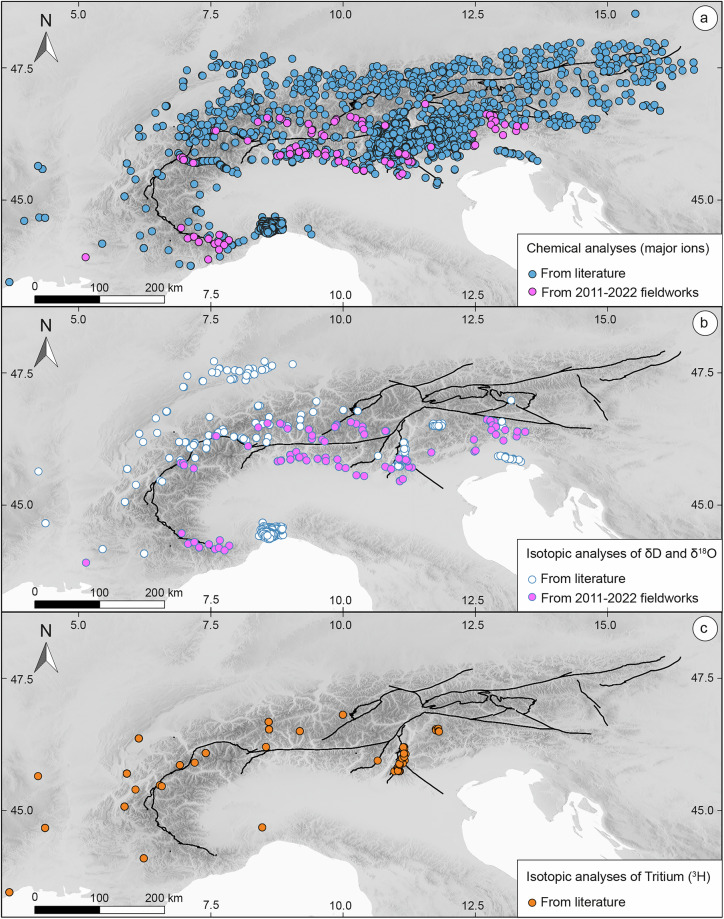
Chemical and water isotope sampling points in the ARETA dataset. (**a**) Chemical analyses of major soluble ions, (**b**) isotopic analyses of δD and δ^18^O, and (**c**) isotopic analyses of Tritium (^3^H). The fuchsia dots in (**a**) and (**b**) represent the sampling points coming from the 2011–2022 fieldworks.

**Fig. 4:**
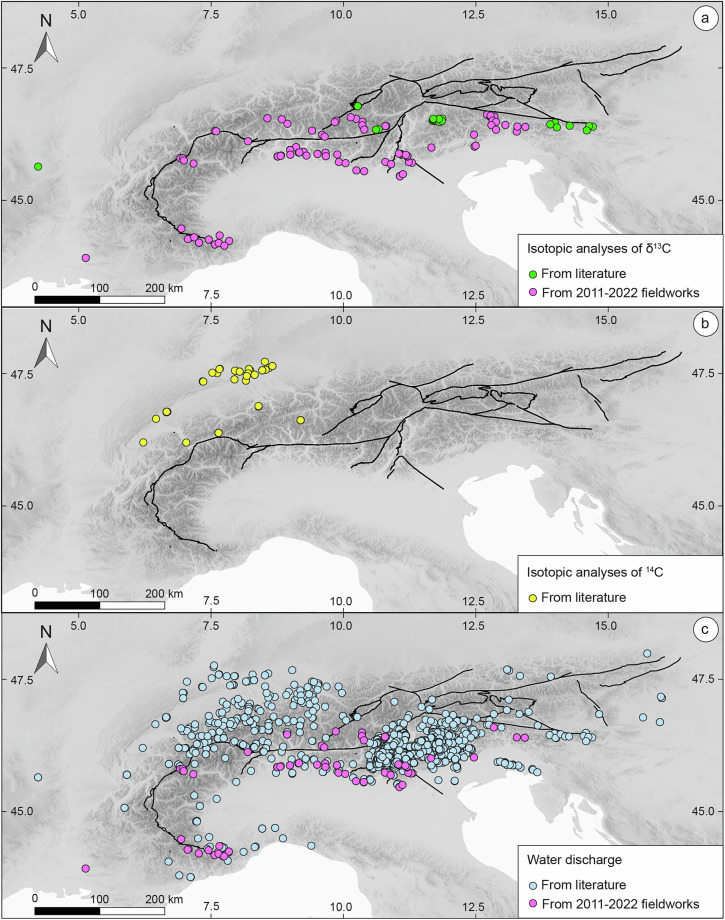
Carbon isotope sampling points and water discharge data in the ARETA dataset. (**a**) δ^13^C and (**b**) ^14^C isotopic analyses. (**c**) Water discharge data. The fuchsia dots in (**a**) e in (**c**) represent the sampling points coming from the 2011–2022 fieldworks.

Figures 3 and 4 illustrate the contribution of fieldwork activities performed by the authors between 2011 and 2022 to the final composition of ARETA dataset. Specifically, the majority of the dataset for major soluble ions (3,374 points) was derived from literature, with only 109 of the 3,381 total sampling points (approximately 3%) having been collected during the new fieldworks (Fig. 3a). Similarly, regarding *Q*, the new fieldwork campaigns contributed 63 measurements, representing a modest ~4% of the 1,481 available data points (Fig. 4c). However, only a clear minority of these 63 data points were measured *in situ* during our campaign; the remaining values were sourced from the literature. Crucially, all 1,481 discharge values utilized represent mean annual flows. This choice is justified by the primary focus on long-term processes at a regional scale, where short-term (e.g., seasonal) hydro-geochemical variability is considered secondary. In contrast, the isotopic parameters saw a more significant boost. For the stable water isotopes (δD and δ^18^O), 106 points were collected during the new fieldworks, which account for approximately 18% of the total 601 analyses (Fig. 3b). The most notable contribution was observed for the *TDIC* data: out of the 139 sampling points with δ^13^C information, a decisive 98 were provided by the new campaign, constituting nearly 70% of the total data for this specific parameter (Fig. 4a). It is worth noting that all available ^3^H and ^14^C data originate exclusively from the literature, as detailed in and Fig. 4b, indicating that the authors’ fieldwork focused primarily on the other listed parameters.

The physical parameters (*Q*, *T*, *pH*, *Eh* and *EC*) for the new sampling points collected by the authors during 2011–2022 fieldworks are presented in Table 8, and the results of the chemical and isotopic analyses are shown in Table 9.

**Table 8:** Location, sampling date and physical parameters of the new sampling points collected by the authors during 2011–2022 fieldworks (*Q*: discharge, *T*: temperature, *Eh*: redox potential, *EC*: electrical conductivity). nd = not determined.

n_DB	Name	Lat	Long	Date	*Q* [L/s]	*T* [°C]	*pH*	*Eh* [mV]	*EC* [μS/cm]
574	Terme di Vinadio Pozzo Stanza	44.289	7.078	25/05/2011	2.3	65.2	7.94	−208	4530
575	Terme di Vinadio Grotta	44.289	7.078	25/05/2011	nd	50.6	8.74	−220	1034
576	Terme di Vinadio Pozzo Parcheggio	44.289	7.078	25/05/2011	nd	54.9	8.65	−270	641
577	Vinadio Fontana Don Bosco	44.276	7.061	25/05/2011	1	7.4	8.18	105	103
578	Vinadio Paese Fontanile	44.308	7.180	25/05/2011	nd	9.8	7.86	190	143
579	Terme di Valdieri Grotta Alta 1	44.207	7.271	26/05/2011	nd	60.9	8.81	−295.5	411
580	Terme di Valdieri Grotta Alta 2	44.207	7.271	26/05/2011	nd	32.1	8.74	−146	208
581	Terme di Valdieri Fontana piazzale	44.207	7.272	26/05/2011	nd	13	8.28	170	73
582	Terme di Valdieri Sorgente casotto	44.206	7.271	26/05/2011	20	12	7.46	215	74
583	Terme di Valdieri Grotta Bassa	44.207	7.271	26/05/2011	nd	56.7	8.88	−258	403
584	Valgesso Fonte Dragonera	44.267	7.455	26/05/2011	250	7.4	7.92	500	197
585	Valgesso Fonte Dragonera 2	44.267	7.455	26/05/2011	nd	7.4	7.94	244	196
586	Bormio Sorgente Cinglaccia	46.493	10.358	22/08/2011	22	38.8	6.9	140	129
587	Sorgenti Adda	46.547	10.239	22/08/2011	nd	4.1	8.2	305.4	25
588	Cepine Fontanile	46.426	10.357	22/08/2011	0.13	12.5	8.14	263.6	15
589	Fontanaccia	46.345	10.393	22/08/2011	2.5	6.1	8.06	201.2	12
590	Samedan Mineralbad	46.534	9.870	23/08/2011	nd	13.2	7.94	126	121
591	Celerina Fontanile	46.513	9.862	23/08/2011	0.07	10.4	7.89	218	18
592	St. Moritz	46.484	9.836	23/08/2011	nd	11.7	6.07	105.7	201
593	S. Michele Valviera	46.577	10.138	23/08/2011	nd	6.7	8.12	−231.3	38
594	Bagni del Masino	46.245	9.598	24/08/2011	63	37.6	8.94	80.5	715
595	Sorgente Valle di Sasso Bisolo	46.211	9.644	24/08/2011	2.5	7.5	8.61	264.1	138
596	Merette A	46.269	9.400	24/08/2011	nd	14.3	6.78	142.6	191
597	Merette B	46.256	9.400	24/08/2011	nd	16.6	7.41	208.1	198
598	Merette C	46.260	9.402	24/08/2011	nd	16.5	7.6	228.4	191
599	Chiavenna Fons Paradisi	46.324	9.406	24/08/2011	nd	14.3	7.54	243.2	199
600	Belvedere Acque Rosse	46.440	9.345	25/08/2011	nd	9.3	7.94	244.6	218
601	Acquacalda	46.538	8.835	25/08/2011	nd	5.9	8.12	259	238
602	Sorgenti Brenna	46.552	8.802	25/08/2011	nd	5.3	7.84	235.1	584
603	Sorgenti Ticino A	46.557	8.563	25/08/2011	nd	16.7	8.28	203.8	11
604	Sorgenti Ticino B	46.477	8.388	25/08/2011	nd	14.8	7.53	228	27.6
605	Fortezza troppo pieno	46.816	11.552	16/03/2012	nd	5.8	8.55	nd	94
1186	La Brigue (Notre-Dames des Fontaines)	44.062	7.655	01/08/2012	nd	12	7.1	285	265
1187	Sospel (Fontanile)	43.877	7.450	01/08/2012	nd	9	7.5	352	575
1188	Fontaine de Vaucluse	43.920	5.131	25/07/2012	20,000	13	7.26	332.5	452
1189	Salgesh Paese	46.315	7.569	22/07/2013	nd	10.2	7.45	109.4	256
1190	Salgesh Valle	46.312	7.595	22/07/2013	nd	10.4	7.77	151.6	245
1191	Fontanone di Barasco	45.836	8.753	22/07/2013	90	13.2	7.98	126.6	276
1192	Sorgente Luvinate	45.835	8.759	22/07/2013	55	12	7.72	136.8	404
1193	Sorgente Pietrificante	45.855	8.821	22/07/2013	30	11.8	7.66	175.8	504
1194	Sorgente Fontane Calde	45.847	8.813	22/07/2013	30	23	8.16	134	378
1195	Grotte di Rescio	46.014	9.106	23/07/2013	nd	11.1	8.52	112.7	322
1196	Ca’ del Fere’	45.960	8.992	23/07/2013	nd	9.5	8.55	236.3	295
1197	Sorgente Bossi	45.957	8.994	23/07/2013	nd	10.6	7.92	184.8	267
1198	Paolaccio A	45.870	8.996	23/07/2013	20	11.6	8.18	248	295
1199	Paolaccio D	45.870	8.996	23/07/2013	6	11.1	8.03	234.2	281
1200	Sorgente Menaresta (Sorgente Lambro)	45.924	9.250	23/07/2013	nd	8.9	7.84	293.2	474
1201	Sorgente Pietrificante Achermann	45.853	9.301	24/07/2013	nd	17	8.92	168.2	313
1202	Sorgente Ferrugginosa	45.885	9.510	24/07/2013	nd	10.3	7.84	124.8	1803
1203	Sorgenti Enna	45.883	9.509	24/07/2013	300	8.5	8.74	146.7	252
1204	Nossana	45.873	9.884	25/07/2013	3160	8.6	8.72	255.5	221
1205	Sorgente Acqua Sparsa	45.732	9.913	25/07/2013	500	11	8.83	285.9	363
1206	Sorgente Milesi	45.710	10.039	25/07/2013	225	11.4	8.22	146.6	479
1207	Dossena	45.908	9.662	26/07/2013	300	9.3	8.82	123.2	259
1208	Fontanone di Paitone	45.557	10.391	27/07/2013	200	13.5	7.72	418.4	542
1209	Lago di Cornino	46.229	13.024	05/08/2013	nd	13.1	8.41	183.6	515
1210	Illeggio	46.429	13.057	05/08/2013	nd	8.7	7.84	229.7	841
1211	Sorgenti Piave	46.623	12.710	06/08/2013	nd	7.8	9.09	222.3	181
1212	Danders	46.590	12.774	06/08/2013	nd	10.3	7.86	278	2030
1213	Genzianella	46.584	12.775	06/08/2013	nd	8.6	8.65	415.9	1123
1214	Clevos	46.585	12.788	06/08/2013	nd	10.2	7.88	385.1	92.9
1215	Edelwais	46.588	12.850	06/08/2013	0.22	7.3	8.09	414.6	238
1216	Fleons (Goccia di Carnia)	46.607	12.779	06/08/2013	nd	11.1	8.87	403.9	124
1217	Acqua rossa	46.516	12.816	06/08/2013	nd	9.5	7.7	219.6	2310
1218	Liariis	46.489	12.881	06/08/2013	nd	13.2	7.97	437.9	210
1219	Sorgente Livenza	46.021	12.475	07/08/2013	4000	9.2	8.32	390.3	222
1220	Gorgazzo	46.040	12.497	07/08/2013	nd	11.6	7.58	423.9	296
1221	Marcellino	46.248	12.465	07/08/2013	nd	10.6	8.17	323.8	325
1222	Oltris	46.427	12.794	08/08/2013	nd	10.4	9.15	209.9	352
1223	Sorgente Arzino	46.342	12.881	08/08/2013	nd	6.1	8.7	292.3	195
1224	Sorgenti Torre	46.307	13.273	08/08/2013	nd	8.5	8.92	427.9	206
1225	Borello Superiore	44.173	7.571	18/09/2013	140	7.6	7.81	241	178
1226	Bossea	44.241	7.840	18/09/2013	130	8.4	7.71	420	202
1227	Beinette	44.344	7.661	18/09/2013	1500	10.5	8.67	367.5	334
1229	Tenda	44.173	7.571	18/09/2013	300	7.1	8.95	306.8	370
1230	Pesio	44.200	7.643	19/09/2013	600	5.8	9.56	nd	246
1231	Fuse	44.151	7.751	20/09/2013	1130	5	8.05	nd	203
1232	Maira	44.474	6.938	21/09/2013	930	6.4	7.38	nd	nd
1383	San Pellegrino Terme	45.841	9.666	16/09/2019	1	23.6	7.82	nd	1450
1384	Fonte di Mompiano	45.573	10.244	17/09/2019	120	nd	nd	nd	nd
1385	Sorgente Resensuola	46.005	11.660	17/09/2019	600	nd	nd	nd	nd
1386	Fontanone di Goriuda	46.394	13.435	18/09/2019	1000	nd	nd	nd	nd
1387	Fontana Zannier	46.401	13.285	18/09/2019	0.5	nd	nd	nd	nd
2896	Arvier Area ricreativa St. Antoine	45.702	7.168	16/05/2022	400	12.3	8.14	−59	178.4
2897	Youla	45.787	6.961	17/05/2022	8.3	7.2	7.46	−23	2200
2898	Fonte Rey	45.804	6.922	17/05/2022	10	7.7	7.89	−45	418
2899	Mont Blanc	45.803	6.920	17/05/2022	10	9.9	8.07	−54	228
2900	La Saxe solforosa	45.803	6.968	17/05/2022	2.5	15.6	nd	−268	997
2901	Orrido Pre Saint Didier Ponte Romano	45.761	6.986	17/05/2022	0.15	30.6	nd	nd	1056
2902	Bognanco Fontanile	46.123	8.196	18/05/2022	0.15	11.1	7.02	−6	115.9
2903	Acquarossa Scarico Fiume	46.455	8.941	19/05/2022	1	24.7	7.5	−30	2660
2904	Falchi della Rupe	45.909	9.156	20/05/2022	200	11.1	7.87	−45	347
2905	Tuff	45.912	9.167	20/05/2022	200	10.1	8.19	−59	289
2906	Aril	45.734	10.789	21/05/2022	1500	10.7	8.19	−60	293
2907	Rio Molini	45.690	10.900	21/05/2022	200	12.4	8.16	−53	436
2908	Risorgiva Montorio_Pieve di S. Maria Assunta	45.465	11.064	22/05/2022	2500	13.5	7.78	−41	377
2909	Risorgiva Montorio Fontanon	45.459	11.066	22/05/2022	2500	14	8.14	−60	380
2910	Azzurra Sorgente Camonda	45.718	11.294	23/05/2022	1.15	18.9	7.41	−41	736
2911	Fonte Regina Staro	45.731	11.234	23/05/2022	0.5	10.7	6.05	45	2630
2912	Rocchi	45.869	11.095	24/05/2022	700	15	7.73	−41	386
2913	San Nicolo	45.871	11.097	24/05/2022	0.2	15.7	8.03	−53	315
2914	Fontana Vecia Noriglio Peschiera	45.885	11.067	24/05/2022	0.3	13.9	7.93	−48	473
2915	Spino	45.889	11.047	24/05/2022	0.15	12	8.09	−55	247
2916	Giordano	45.501	11.122	24/05/2022	5	9.8	8.45	−63	391
2917	Borcola	45.832	11.205	24/05/2022	0.2	10.9	8.09	−55	397
2918	Ertile Bassa	45.843	11.207	24/05/2022	0.2	8	8	−51	310
2919	Acqua Negra	45.865	11.178	24/05/2022	400	10.7	8.14	−57	296
2920	Terme di Rabbi	46.408	10.808	25/05/2022	1	9.7	5.97	nd	1937
2921	Rabbi Fontanile	46.410	10.793	25/05/2022	0.2	11.6	7.74	−38	95.1

**Table 9:** Chemical (major soluble ions) and isotopic composition (δD, δ^18^O, and δ^13^C) of the new sampling points collected by the authors during 2011–2022 fieldworks. nd = not determined.

n_DB	Ca^2+^[mg/L]	Mg^2+^ [mg/L]	Na^+^ [mg/L]	K^+^ [mg/L]	HCO_3_^-^ [mg/L]	SO_4_^2+^ [mg/L]	Cl^-^ [mg/L]	δD [‰]	δ^18^O [‰]	δ^13^C [‰]
574	78.3	0.15	726.2	38.9	23.18	31.36	1250	−90.87	−12.69	−16.96
575	5.06	0.03	193.68	7.5	49.11	30.48	209.3	−89.34	−12.88	−7.36
576	5.71	0.21	74.6	4.69	57.34	22.82	100.11	−91.11	−13	−9.28
577	14.51	0.66	2.35	0.36	46.21	8.94	0.59	−89.42	−12.79	−5.42
578	16.3	1.95	1.87	0.09	28.21	32.77	0.29	−94.03	−13.46	−17.42
579	4.38	0.03	74.6	2.48	52	55.17	25.44	−86.17	−12.28	−8.15
580	11.2	0.8	24.56	1.54	54.6	28.60	8.57	−84.42	−11.81	−13.27
581	4.1	0.28	7.33	0.2	16.32	9.85	2.8	−92.14	−12.88	−23.91
582	4.2	0.29	7.21	0.3	19.52	9.75	2.6	−92.2	−12.94	−20.34
583	4.6	0.02	70.6	2.3	54.29	56.47	25.38	−85.69	−12.2	−8.77
584	33.65	2.51	0.67	0.06	112.39	1.91	0.55	−92.64	−13.38	−7.86
585	33.53	2.55	0.32	0.04	126.73	1.84	0.18	−94.66	−13.32	−8.03
586	200.4	54.7	17.9	4.18	172.94	540.21	5.27	−98.16	−13.59	−5.04
587	34	7.74	0.38	0.16	128.1	22.66	0.16	−100.45	−13.88	−6.04
588	18.12	2.55	2.54	1.79	41.79	26.63	0.28	−87.36	−12.55	−7.75
589	13.5	2.74	2.3	2.84	40.41	16.45	0.18	−83.81	−11.95	−6.32
590	190	7.06	61.7	4	62.53	517.58	14.84	−106.33	−14.49	nd
591	19.08	7.54	0.79	0.28	80.52	24.66	0.18	−109.44	−14.8	−8.37
592	268.8	32.88	169.4	3.74	1248.06	168.06	20.15	−99.23	−13.47	−1.18
593	45.7	14.12	7.22	1.03	233.63	17.03	0.36	−104.95	−14.11	−9.51
594	31.1	0.09	108.2	3.58	26.03	224.91	19.65	−86.36	−12.71	−18.75
595	16.36	1.9	2.76	3.08	34.47	27.91	0.52	−70.74	−10.44	−8.6
596	20.1	5.29	4.34	3.14	70.52	22.42	2.56	−66.67	−9.76	nd
597	24.9	3.65	3.68	2.64	61.98	30.38	2.29	−67.08	−10.11	nd
598	23.1	3.43	3.54	4.26	58.56	32.26	2.27	−69.56	−9.93	nd
599	27.1	2.76	1.62	1.51	41.07	48.26	0.66	−69.05	−10.23	−9.29
600	444	31.56	2.98	2.1	109.59	1203.96	2.23	−81.97	−11.91	nd
601	32.8	6.25	0.52	4.7	138.47	18.56	0.21	−82.68	−12.2	−9.27
602	74.9	17.8	0.67	1.11	82.96	204.92	0.27	−83.7	−12.13	nd
603	1.72	0.14	0.41	1.42	6.56	2.59	0.27	−48.9	−7.39	−13.78
604	3.3	0.11	1.95	0.62	11.13	1.31	2.22	−73.57	−10.03	nd
605	14.4	0.88	1.79	1.05	49.5	3.89	1.08	nd	nd	nd
1186	40.4	2.64	2.3	0.39	134.81	8.16	0.99	nd	nd	nd
1187	97	1.56	3.45	0.39	290.97	8.16	4.39	nd	nd	nd
1188	82.4	5.16	2.99	0.94	261.69	12.48	4.6	−53.11	−7.95	−12.53
1189	41.42	6.52	0.92	3.63	123.19	26.95	3.1	−110.48	−14.91	−7.7
1190	39.12	5.01	1.3	0.92	93.92	38.55	1.45	−112.96	−15.08	−6.83
1191	45.21	6.42	1.96	0.42	169.84	5.73	2.36	−57.79	−8.68	−12.11
1192	60.98	9.67	5	2.52	220.76	8.94	11.87	−57.09	−8.56	−13.02
1193	58.98	30.96	1.29	0.01	356.15	5.54	1.49	−56.37	−8.6	−12.83
1194	45.41	18.5	3.04	0.55	246.38	4.81	6.15	−58.42	−8.96	−12.69
1195	57.78	6.1	1.4	0.01	211	5.23	2.19	−54.93	−7.62	−11.62
1196	53.49	3.89	1.96	1.83	176.85	4.61	5.24	−60.01	−9.05	−11.83
1197	47.21	4.81	1.07	0.01	164.66	3.69	1.11	−59.33	−8.94	−12.23
1198	52.2	4.71	2.16	0.52	189.05	7.65	3.64	−58.08	−8.82	−12.83
1199	50.7	4.22	1	0.12	185.39	7.12	1.94	−57.96	−8.8	−12.86
1200	69.26	15.24	7.05	0.23	284.19	10.29	8.57	−61.05	−9.07	−13.57
1201	55.59	6.63	1.71	0.2	185.39	7.91	1.81	−56.9	−8.56	−9.76
1202	331.74	51.17	5.34	2.19	148.8	851.59	1.37	−60.02	−9.11	−6.45
1203	34.73	11.38	0.55	0.01	150.02	3.29	0.73	−60	−8.97	−10.56
1204	34.93	6.8	0.5	0.22	130.51	8.33	0.86	−64.28	−9.48	−8.61
1205	66.57	4.36	2.95	0.45	213.44	6.92	5.52	−58.27	−8.53	−12.93
1206	91.02	5.68	2.64	0.71	306.14	7.49	2.81	−55.42	−8.25	−14.11
1207	38.52	8.35	1	0.01	128.07	21.24	1.06	−64.93	−9.62	−6.52
1208	85.13	15.18	5.94	1.12	335.41	10.55	8.53	−56.65	−8.31	−14.05
1209	77.74	15.38	2.7	0.27	194.85	121.84	2.56	−59.99	−8.86	nd
1210	129.74	25.12	1.5	0.32	251.37	259.23	0.93	−51.56	−7.32	−10.57
1211	32.93	2.08	0.32	0.01	138.33	5.50	0.25	−91.76	−12.74	−6.17
1212	378.44	55.61	3.76	0.8	252.86	981.40	1.9	−65.34	−9.52	−6.57
1213	183.03	36.72	3.17	0.42	218.28	414.92	1.79	−63.95	−9.48	−7.51
1214	11.28	2.41	2.82	0.22	48.34	10.77	0.2	−64.71	−9.42	−17.57
1215	45.61	1.3	1.24	0.01	181.71	2.55	0.24	−64.81	−9.4	−12.67
1216	18.66	3.57	1.12	0.01	91.85	1.94	0.15	−79.77	−11.3	−9.57
1217	515.97	60.44	1.94	0.01	200.06	1162.16	0.52	−63.58	−9.3	−5.22
1218	26.55	9.55	0.75	0.01	106.35	2.44	0.41	−63.53	−9.54	−10.28
1219	39.72	3.42	0.82	0.01	138.83	2.66	0.95	−60.08	−9.14	−9.52
1220	50.3	4.3	1.15	0.01	189.27	2.78	1.66	−53.13	−8.2	−11.06
1221	37.03	18.18	0.42	0.01	214.56	5.67	0.36	−59.44	−8.89	−11.94
1222	45.01	13.41	1.08	0.01	153.2	44.85	0.5	−60.47	−8.82	−8.81
1223	30.74	5.6	0.39	0.01	127.17	1.66	0.4	−58.39	−8.95	−8.17
1224	27.94	8.02	0.88	0.01	132.38	1.95	1.15	−54.77	−8.46	−8.77
1225	30.24	3.71	0.82	1.39	109.7	2.18	0.41	−75.56	−10.91	−11.58
1226	37.33	2.43	0.91	0.19	107.09	2.20	1.53	−78.8	−10.92	−11.91
1227	49.3	8.68	2.71	0.42	137.96	47.98	3.37	−82.49	−11.58	−11.21
1229	62.97	5.63	1.49	0.01	129.78	71.73	0.82	−82.17	−11.66	−11.07
1230	38.82	6.16	0.43	0.01	101.89	28.71	0.31	−78.36	−11.07	−7.11
1231	34.23	3.79	0.33	0.01	124.94	7.04	0.25	−74.58	−10.46	−7.83
1232	88.92	23.03	4.27	0.51	183.6	181.72	4.38	−91.89	−12.61	−6.32
1383	196.4	48.7	42.36	2.14	234.24	436.32	66.52	−60.85	−9.14	−7.36
1384	80.95	23.52	6.82	1.07	377.9	9.18	10.23	−54.75	−8.3	−13.85
1385	49	10.7	2.33	0.59	204.96	16.30	2.44	−66.04	−9.71	−10.57
1386	33.3	2.28	0.4	0.06	118.34	1.91	0.35	−58.53	-9	−2.23
1387	33.9	16	0.73	0.15	218.99	2.74	0.64	−58.74	−8.85	−10.76
2896	25.1	3.45	2.33	0.59	74.42	14.58	2.3	−101.95	−14.01	−6
2897	411.2	43.24	0.97	1.59	155.67	1003.61	0.76	−103.41	−14.16	−5.95
2898	61.6	5.24	1.79	2.12	99.31	84.98	0.55	−106.01	−14.28	−9.33
2899	29.1	2.18	1.79	1.9	62.71	33.28	0.64	−105.69	−14.27	−10.05
2900	130.4	7.56	55.2	3.2	422.85	79.35	45.57	−113.59	−15.35	−7.41
2901	57	14.88	62	2.98	352.82	145.83	40.48	−108.2	−14.53	−5.4
2902	13.7	0.79	1.62	0.52	44.16	4.98	0.3	−69	−10.16	−12.15
2903	450	65.5	23.7	13.4	604.88	964.43	4.44	−73.3	−10.6	−1.84
2904	50.8	5.8	2.23	0.4	190.32	5.68	4.9	−54.59	−8.63	−12.04
2905	44.7	3.74	0.69	0.09	168.6	3.22	0.6	−56.88	−8.94	−11.53
2906	39.5	4.86	0.78	0.5	156.65	2.01	1.63	−62.94	−9.48	−9.1
2907	45.2	5.74	1.11	0.54	180.07	9.00	1.38	−63.68	−9.57	−9.78
2908	51.1	8.5	2.86	1.21	199.35	9.14	5.28	−60.5	−9.09	−10.83
2909	49.1	8.42	2.84	1.33	199.35	9.17	5.08	−60.32	−9.11	−10.21
2910	79.7	30.52	1.4	1.03	239.36	155.73	0.93	−53.75	−8.55	−10.31
2911	158.8	90.7	300	8.45	1862.13	79.26	8.63	−55.05	−8.73	−2.35
2912	50	8.83	2.7	0.36	211.3	6.66	2.85	−64.28	−9.42	−10.94
2913	42.3	7.37	1.16	0.88	180.56	3.41	0.9	−64.34	−9.33	−10.4
2914	72.7	5.14	3.63	0.56	251.32	6.83	11.62	−67.34	−9.66	−11.02
2915	30.5	7.75	0.56	0.16	153.96	5.89	0.62	−69.74	−10.47	−7.62
2916	31.3	21.12	0.61	0.19	232.04	1.78	0.7	−62.51	−9.58	−9.11
2917	30	11.04	1	0.91	180.07	1.13	3.95	−61.21	−9.43	−11.26
2918	30.7	14.36	0.4	0.15	200.57	1.67	0.59	−62.94	−9.64	−10.41
2919	38.7	7.37	1.07	0.23	178.36	2.89	2.02	−66.17	−9.84	−9.41
2920	90.9	27.8	389	28.25	1904.83	12.71	174.64	−93.23	−12.66	−0.5
2921	11	1.66	3.64	1.07	31	14.95	0.22	−90.29	−12.59	−9.34

### Technical Validation

To evaluate the completeness of ARETA, we elaborated an UpSet plot to display the intersections of its multiple parameter sets.

**Fig. 5:**
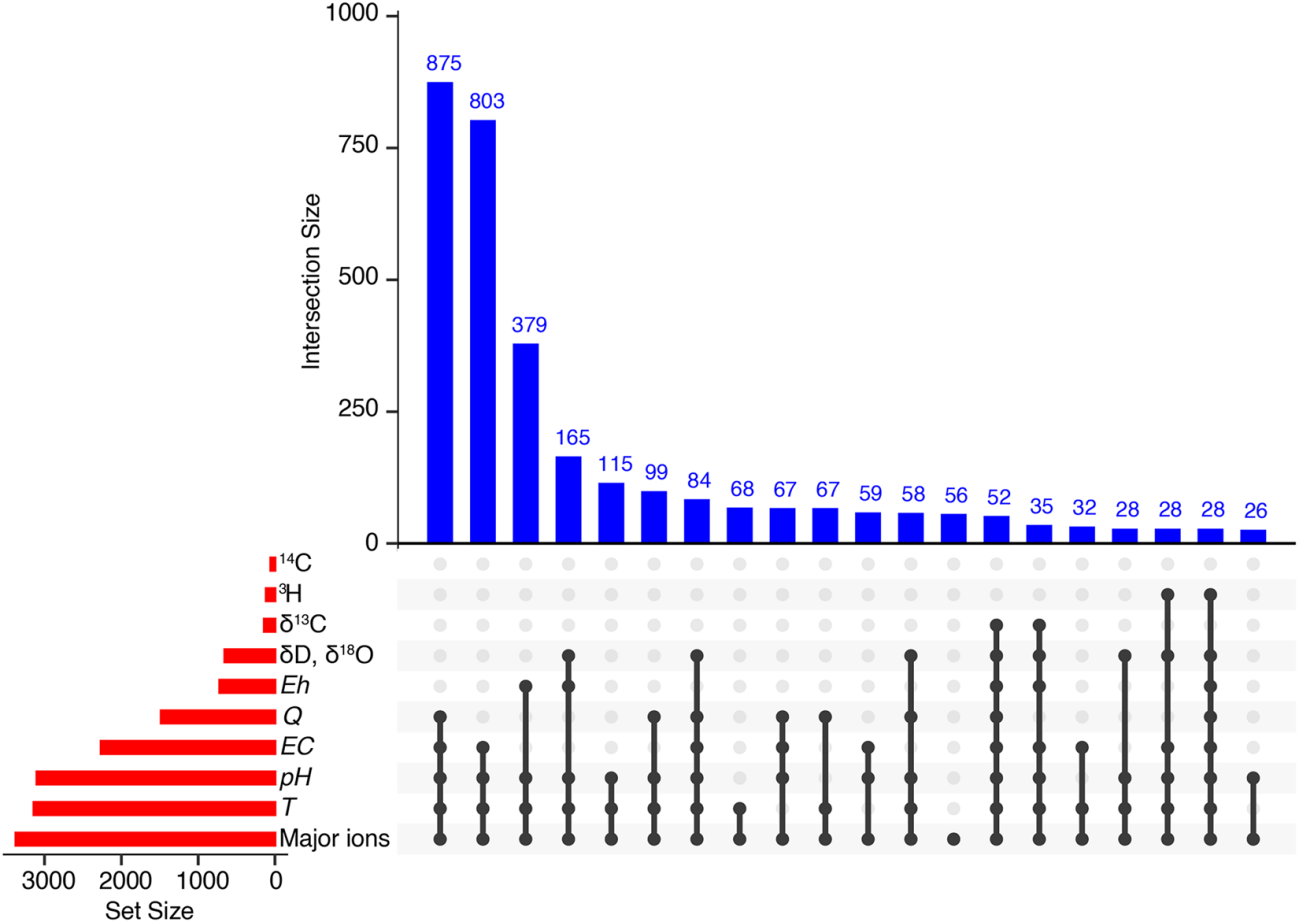
Data hierarchy and parameter intersections in the ARETA dataset. UpSet plot showing the distribution of hydrogeochemical parameters. Horizontal red bars indicate the total sampling points per parameter. The bottom matrix (dots and lines) defines the parameter combinations, while vertical blue bars quantify the cardinality (number of points) of each unique intersection.

Figure 5 presents the UpSet plot illustrating the co-occurrence of parameters in the dataset. The red horizontal bars on the left represent the individual size of each set, showing the total number of sampling points containing each parameter. The dots and connecting black lines constitute the intersection matrix, where each column represents a unique set intersection (combination). A black dot indicates that the corresponding parameter is present within that specific combination. Finally, the blue vertical bars display the cardinality (or size) of each intersection, quantifying the number of sampling points that belong to that exact combination of parameters.

The plot highlights that the largest intersection, with 875 records, corresponds to the co-occurrence of *Q*, *EC*, *pH*, *T*, and major ions, as indicated by the connected dots in the intersection matrix. This combination represents the most commonly available suite of fundamental hydrogeochemical data. The next largest intersection, with 803 records, involves *EC*, *pH*, *T*, and major ions, that corresponds to the previous combination excluding *Q*. Moreover, the plot highlights that the intersections involving the environmental isotopes δ^13^C, δD, δ^18^O are significantly smaller. For example, the combined availability of δ^13^C, δD, δ^18^O, *Q*, *EC*, *pH*, *T*, and major ions yields only 58 records, highlighting the limited comprehensive sampling of both major ions and isotopes together.

The plot clearly demonstrates a data hierarchy: *Q*, *pH*, *T*, *EC*, and major ions are frequently measured together, forming the largest core dataset (875 records). Conversely, variables such as *Eh* and isotopic parameters (δ^13^C, δD, δ^18^O, ^3^H, ^14^C) are measured less often and rarely co-occur with a complete suite of all other variables. This distribution reflects typical sampling priorities in hydrogeochemical studies.

Before performing the technical validation, we assessed the spatial coverage of the data. Figure 2a highlights that the sampling points containing complete analyses of major soluble ions cover quite efficiently the entire Alpine region, while Figs. 2b,2c, 3a show that sampling points with data on isotopic composition of water (δD, δ^18^O, and ^3^H) and of ^13^C, the cover with discrete efficiency almost all the Alpine region, with the exception of its North-Eastern sector. With respect to radiocarbon ^14^C, Fig. 3b shows that among the sampling points for which isotopic composition is available, only about ten are located in a small portion of the north-western sector of the Alpine region, while the remaining ones are distributed across the Jura Mountains and the Molasse Basin (see Fig. 1). Water discharge data is available for fewer than 50% of the sampling points, with the data scarcity being most pronounced in the north-eastern sector of the Alps (see Fig. 3c). Despite their critical importance for water resource management, reliable long-term discharge (flow rate) data for springs are often scarce or unavailable in public databases. This clearly constitutes a shortcoming of the dataset, highlighting that Alpine region represents an area where future efforts need to be concentrated to fill this data gap.

ARETA technical validation was performed aiming at verifying the presence of duplicates in the dataset as well as errors in the chemical analyses. The first issue was verified checking coordinate duplicates in the *DB_location.shp* vector file. This procedure highlights the presence of 698 duplicates, representing ~18% of the total sampling points. Among these, only 34 (i.e. less than 1%) share the same record in the “Name_DB” column. A detailed review of the reference documents used to populate the dataset reveals that, apart from the 34 aforementioned sampling points, the remaining duplicates correspond to location georeferenced only at municipality accuracy.

**Fig. 6:**
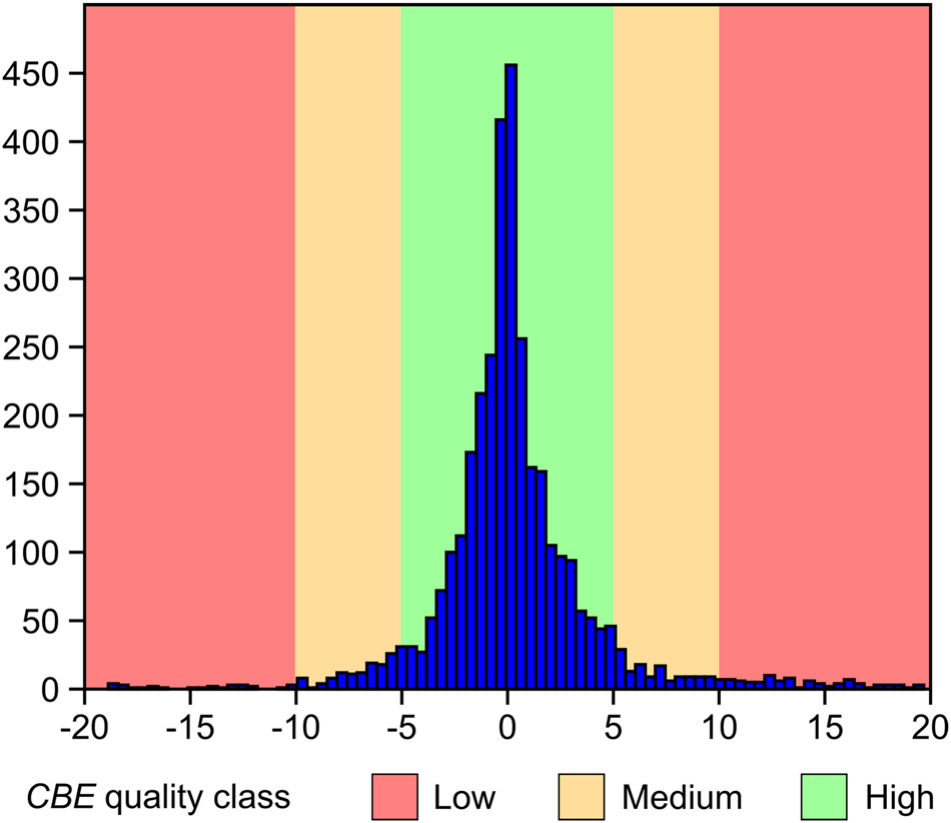
Histogram of Charge Balance Error (*CBE*) of the 3374 sampling points containing chemical analyses of major soluble ions. The three colours represent the quality classes of *CBE* (i.e. low, medium, high). Histogram was elaborated with PAST 2.07^101^.

The analytical uncertainty of the chemical analyses of major soluble ions in the dataset has been evaluated by checking the charge balance error (*CBE*) verifying if the total sum of all positive charges (cations) are equal to the total sum of all negative charges (anions) by using the following equation:

1 $${CBE}=[(\Sigma {C}_{{cat}}-\Sigma {C}_{{an}})/(\Sigma {C}_{{cat}}+\Sigma {C}_{{an}})]\times 100$$

where *C*_*cat*_ and *C*_*an*_ are, respectively, the cations and anions concentration expressed in eq/L. 1 To evaluate the quality of the analyses, we considered three classes based on the absolute value of *CBE*: high, where |*CBE*| is between 0 and 5%; medium, where |*CBE*| is between 5% and 10%; and low, where |*CBE*| is greater than 10%^28^. Figure 6 shows the *CBE* histogram, highlighting the three classes indicating the quality of the chemical analyses. The histogram shows that ~88% of the sampling points have high quality analyses, with |*CBE*| ≤ 5%, while ~8% have medium quality, and ~4% low quality. Although 123 samples (~4% of the total dataset) exhibit a low-quality *CBE* values, these literature data were retained. Their inclusion is justified by their potential exploratory value, as these outlier analyses may highlight areas where analytical effort should be concentrated in future fieldwork campaigns to better characterize the hydrogeochemical regime, even if they require cautious use in quantitative modelling.

Regarding the uncertainty of the isotopic analyses, unfortunately the documents considered to populate ARETA do not contain this kind of information. On the contrary, δD, δ^18^O, and δ^13^C measurements in the samples collected by the authors between the 2011–2022 fieldwork activities include the standard deviation (*σ*) of the analysis. To quantify the uncertainty of this sub-set, we included the associated coefficient of variation (*CV*) calculated following Eq. 2

2 $${CV}=(\sigma /|\mu |)\times 100$$

where |*μ*| is the absolute value of the analytical measurement. Similarly to the *CBE* classification, we established three *CV* classes for data quality: high (*CV* ≤ 5%), medium (5% < *CV* ≤ 10%), and low (*CV > *10%). Table 10 summarizes the minimum, maximum, and average *CV* values for the isotopic parameters δD, δ^18^O, and δ^13^C (*CV_δD*, *CV_δ*^*18*^*O*, and *CV_δ*^*13*^*C*, respectively) derived from the 2011–2022 measurements performed by the authors. *CV* for δD (representing the relative analytical precision) ranges from 0% to 5.03%, with a mean value of 1.66%. Similar levels of precision are observed for δ^18^O, where the *CV* ranges from 0% to 5.56% and the mean value is 1.96%. In contrast, δ^13^C shows a significantly broader range of *CV* values, spanning from 0% to 38.05%, with a mean of 1.36%. However, upon closer inspection, only three samples exhibit extremely high *CV* values (14.88%, 20.52%, and 38.06%). Excluding these three outliers, the *CV_δ*^*13*^*C* range narrows considerably, from 0% to 5.98%, and the mean precision improves to 0.63%.

**Table 10:** Minimum, maximum and average coefficient of variation (*CV*) values for δD, δ^18^O, and δ^13^C measurements (named as *CV_δD*, *CV_δ18O*, and *CV_δ13C*, respectively).

	*CV_*δD	*CV_*δ^18^O	*CV_*δ^13^C
**Min**.	0%	0%	0%
**Max**.	5.03%	5.56%	38.06%
**Avg**.	1.66%	1.96%	1.36%

Examining the *CV* quality classes across the isotopic analyses, the majority of samples show high quality (*CV* ≤ 5%). Out of the 57 samples analysed for δD, only one falls into the medium quality class (with a *CV* value of 5.03%). A similar distribution was observed for the 57 δ^18^O analyses, where six samples were classified as medium quality. Among the 98 δ^13^C analyses, one sample falls into the medium quality class and three samples were categorized as low quality (*CV* > 10%). All remaining values for the three isotopes are classified as high quality. Following the approach for spring waters with |*CBE*| > 10%, we decided to maintain the three δ^13^C analyses with low quality for their potential exploratory value, especially for two of them, which are, respectively, sulfidic and CO_2_-rich springs.

**Fig. 7:**
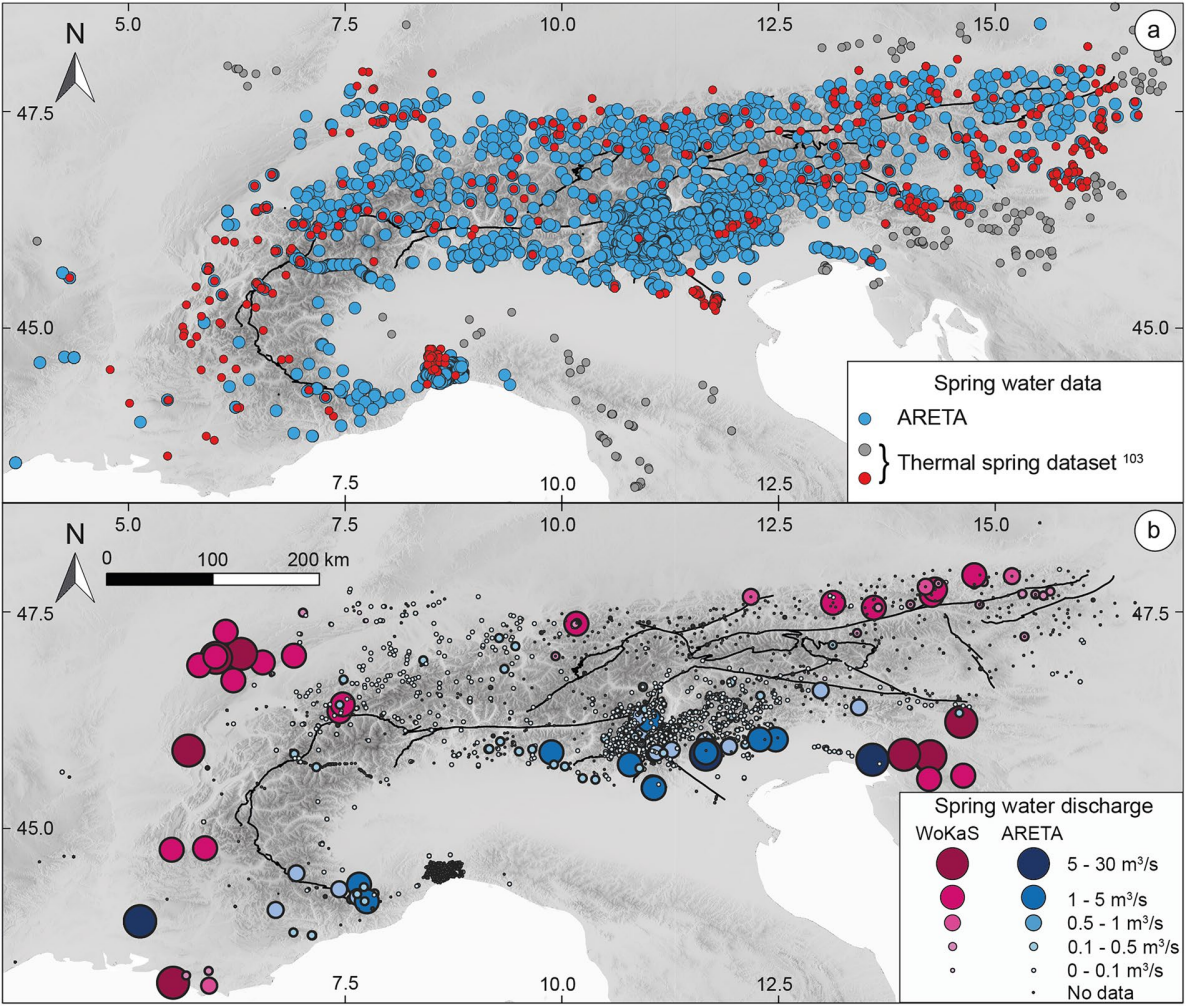
Comparison of ARETA dataset with other published datasets. (**a**) ARETA sampling points compared with the Alpine thermal spring dataset^29^. The combined grey and red dots represent the complete dataset, while red dots represent those falling in the Alpine area. (**b**) ARETA sampling points compared with those of WoKaS dataset^20^ classified according to water discharge.

ARETA was finally compared with a published Alpine thermal spring dataset^19,29^, and with the World Karst Spring (WoKaS) dataset^20^. The results of this comparison are illustrated in Fig. 7.

We firstly analysed the overlap between ARETA and the 946 records reported Alpine thermal spring dataset^29^. As shown in Fig. 7a, ∼22% of the full dataset (red and grey dots) overlaps with ARETA (blue dots). However, when restricted to the 243 points located within the Alpine area (red dots only in Fig. 7a), the overlap significantly increases to ∼48%.

The comparison with the WoKaS dataset^20^ (see Fig. 7b) reveals complementary spatial coverage differences. ARETA excludes most karst springs in the Northern Alpine sector, while the WoKaS dataset, conversely, lacks most Italian karst springs^30^.

This comparative analysis underscores the inherent challenge of compiling a truly comprehensive dataset encompassing physical, chemical, and isotopic information across large geographical areas. The observed differences in spatial coverage and content across datasets highlight their complementary nature. ARETA significantly contributes to the existing body of knowledge by filling key geographic and hydrogeochemical gaps within the Alpine chain. Therefore, while ARETA currently represents a valuable and extensive resource for the region, it is best utilized in conjunction with other established databases to maximize data coverage for broad-scale studies. Consequently, further refinement and database integration efforts are warranted to fully realize a comprehensive hydrogeochemical overview of the Alpine chain.

## Data Availability

The dataset is available at 10.6084/m9.figshare.29850941.

## Code availability

No custom code was employed by the authors in this work
