# Correction: ARETA (Alpine caRbon cyclE daTAset): a dataset on physical, chemical and isotopic data of Alpine groundwaters

**DOI:** 10.1038/s41597-026-07601-9

**Published:** 2026-06-13

**Authors:** Marco Donnini, Laura Melelli, Marino Vetuschi Zuccolini, Federica Fiorucci, Carlo Cardellini, Francesco Frondini

**Affiliations:** 1 CNR-IRPI (Consiglio Nazionale delle Ricerche – Istituto di Ricerca per la Protezione Idrogeologica), Perugia, Italy; 2 Università degli Studi di Perugia – Dipartimento di Fisica e Geologia, Perugia, Italy; 3 Università di Genova – Dipartimento di Scienze della Terra, dell’Ambiente e della Vita, Genova, Italy

Correction to: *Scientific Data* 10.1038/s41597-025-06541-0, published online 24 March 2026

In Eq. 1 of this article, the equation was incorrectly given as

$${CBE}=\left[(\sum {C}_{{cat}}\,-\,\sum {C}_{{an}})/(\sum {C}_{{cat}}\,-\,\sum {C}_{{an}})\right]\times 100$$

The correct equation is

$${CBE}=\left[(\sum {C}_{{cat}}-\sum {C}_{{an}})/(\sum {C}_{{cat}}+\sum {C}_{{an}})\right]\times 100$$

The original article has been updated.

